# The Effect of Phase Separation on the Mechanical Behavior of the Co–Cr–Cu–Fe–Ni High-Entropy Alloy

**DOI:** 10.3390/ma14216523

**Published:** 2021-10-29

**Authors:** Heling Liu, Chuanxiao Peng, Xuelian Li, Shenghai Wang, Li Wang

**Affiliations:** 1 School of Mechanical, Electrical & Information Engineering, Shandong University (Weihai), Weihai 264209, China; liuhelingfight@163.com (H.L.); shenghaiw@163.com (S.W.); wanglihxf@sdu.edu.cn (L.W.); 2 Weihai Institute of Industrial Technology, Shandong University, Weihai 264209, China

**Keywords:** high-entropy alloys, phase separation, mechanical behaviors, molecular dynamics simulation

## Abstract

Phase separation phenomena in high-entropy alloys (HEAs) have attracted much attention since their discovery, but little attention has been given to the dynamics of the deformation mechanism of this kind of HEA during uniaxial tension, which limits their widespread and practical utility. In this work, molecular dynamics simulation was used to study the effect of phase separation on the mechanical properties of an HEA under uniaxial tensile loading. Moreover, the associated deformation behavior of the Co–Cr–Cu–Fe–Ni HEA was investigated at the nanoscale. Models with Cu-rich grain boundaries or grains were constructed. The results showed that Cu-rich grain boundaries or grains lowered the strength of the Co–Cr–Cu–Fe–Ni HEA, and Cu-rich grain boundaries significantly reduced ductility. This change of mechanical properties was closely associated with a deformation behavior. Furthermore, the deformation behavior was affected by the critical resolved shear stress of Cu-rich and Cu-depleted regions and the uneven stress distribution caused by phase separation. In addition, dislocation slipping and grain boundary sliding were the main mechanisms of plastic deformation in the Co–Cr–Cu–Fe–Ni HEA.

## 1. Introduction

High-entropy alloys (HEAs) contain multiple principal (or major) elements, commonly five or more, in equimolar or near-equimolar ratios, differently from conventional alloys. While the configurational entropy of an alloy increases with the increasing number of alloying elements, multiple principal elements of an HEA lead to high entropy of mixing that thus suppresses the formation of intermetallic compounds [1,2,3]. Therefore, stable solid solution phases, such as face-centered cubic (FCC) phase, body-centered cubic (BCC) phase, and hexagonal close-packed (HCP) phase can be obtained [4,5]. Meanwhile, HEAs have been shown to exhibit outstanding mechanical and chemical properties, such as high strength and hardness [1,6], exceptional ductility, as well as excellent oxidation and corrosion resistances [7], and thus have become potential candidate materials for many challenging industrial applications. The design and application of HEAs require a comprehensive understanding of their properties and underlying deformation mechanisms. Co–Cr–Cu–Fe–Ni, with a simple FCC structure [8], is the earliest discovered quinary high-entropy alloy [9,10] and also one of the most studied high-entropy alloy systems. Al, Nb, Ti, and Zr are specially added to improve the properties of the Co–Cr–Cu–Fe–Ni HEA [11,12,13,14]. Recently, the Co–Cr–Cu–Fe–Ni HEA system has received more and more attention because it undergoes the interesting liquid-phase separation (LPS) phenomenon [15,16,17,18,19].

LPS was studied by Nakagawa in Cu–Co and Cu–Fe binary systems for the first time [20]. Up to now, it has been observed in a variety of materials, including metals, polymers, and ceramics [21,22,23]. For metals, LPS has been reported in Cu–Co [24,25], Cu–Fe [24,26,27], Cu–Cr [28], Cu–Nb [29], Cu–Fe–Co [24,30], Cu–Nb–Ni [31], Ag–Nb–Ni [32], Cu–Cr–Si [33], Fe–Cu–Si [26], and other binary and ternary alloy systems. LPS in HEAs was first discovered by Hsu et al. in 2007 with a study of the alloying behavior of Al–Co–Cr–Cu–Ni-based HEAs after the addition of Fe, Ag, and Au [34], suggesting that it exists not only in the binary or ternary alloy systems mentioned above but also in HEAs. Recently, Derimow et al. investigated the solidification microstructures of equiatomic Co–C–Cu with added Fe, Mn, Ni, V, Fe–Mn, Fe–Ni, Fe–V, Mn–Ni, Mn–V, and Ni–V to the composition. It was found that only three of the alloys exhibited dendritic solidification (Co–Cr–Cu–Ni, Co–Cr–Cu–Fe–Ni, and Co–Cr–Cu–Mn–Ni), while the remaining combinations underwent stable LPS [35]. Munitz et al. suggested an additional mechanism of LPS due to the substantially larger attractive interactions between Ni and Al compared to the other binaries in Al–Cr–Fe–Ni–Mo_0.3_ HEA [36]. As for the Co–Cr–Cu–Fe–Ni HEA system, the LPS phenomenon was found in as-cast Co–Cr–Cu–Fe_0.5_–Ni HEA [19]. This suggests that LPS also exists in Co–Cr–Cu–Fe–Ni HEA. Liu et al. further investigated the LPS phenomenon in Co–Cr–Cu–Fe_x_–Ni HEAs. The results showed that LPS occurs when the melt undercooling exceeds a critical undercooling. Meanwhile, many Cu-rich nanosized grains were also observed in the Cu-rich matrix [15]. Wang et al. also verified LPS and characterized a rapid dendritic growth phenomenon in Co–Cr–Cu–Fe–Ni HEA [17]. Guo et al. studied LPS in Co–Cr–Cu–Fe–Ni HEA by the supercooling method. The results showed that the yield strength and elongation of equiatomic Co–Cr–Cu–Fe–Ni alloy decreased significantly due to the inhomogeneity of the microstructure resulting from LPS [18]. Verma et al. studied the effect of Cu on microstructure evolution and phase formation in Co–Cr–Cu_x_–Fe–Ni HEA systems. It was found that Cu segregated at grain boundaries [16]. In these studies, LPS in Co–Cr–Cu–Fe–Ni HEA and its effect on mechanical properties were studied on a macroscale. However, the effect of phase separation on deformation behaviors was not studied. At the nanoscale, the molecular dynamics (MD) simulation performed by Tian et al. found that partial dislocation slipping dominate the initial plastic deformation of Co–Cr–Cu–Fe–Ni HEA [37]. Liu et al. performed an MD study of the temperature dependence of the mechanical properties and plastic inception of the Co–Cr–Cu–Fe–Ni high-entropy alloy [38]. However, neither of these two MD simulations involved experimentally confirmed phase separation.

As shown by the above-mentioned studies, the relationship between composition segregation and deformation behavior of the Co–Cr–Cu–Fe–Ni HEA at the nanoscale is still largely unknown, and more research is needed. However, it is experimentally challenging to directly observe the deformation behavior of the Co–Cr–Cu–Fe–Ni HEA with nanoscale resolution, while MD simulation can provide deep insights into the atomic deformation mechanism of the nanoscale Co–Cr–Cu–Fe–Ni HEA. In other words, MD simulation seems to be one of the very few methods available to capture the dynamic deformation process in the stress–strain responses of the Co–Cr–Cu–Fe–Ni HEA. In this work, MD simulation was employed to investigate the effect of composition segregation on the deformation behavior of the Co–Cr–Cu–Fe–Ni HEA. Additionally, the mechanical properties and deformation mechanism of the Co–Cr–Cu–Fe–Ni HEA are discussed in detail. This theoretical investigation of phase separation influence on the deformation behaviors of Co–Cr–Cu–Fe–Ni HEA may inspire new ideas to improve its mechanical properties.

## 2. Simulation Method

**Figure 1:**
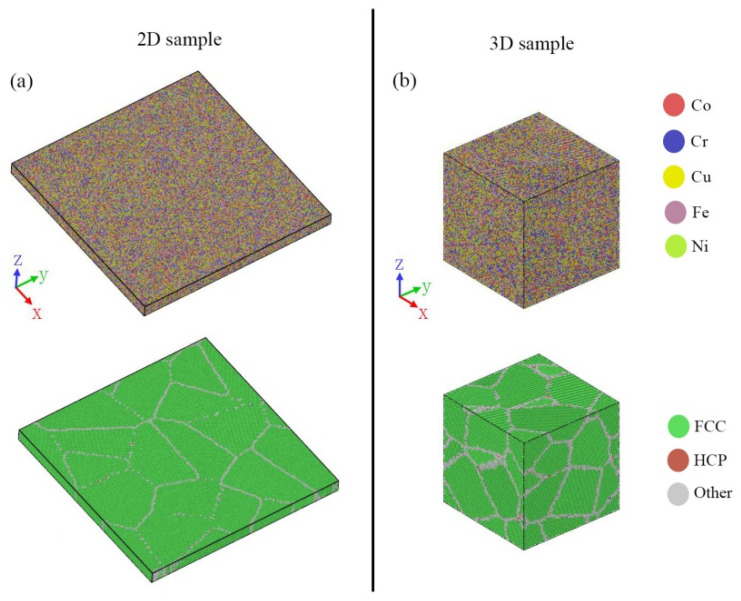
Atomistic model of the Co–Cr–Cu–Fe–Ni HEA sample with random composition distribution (RCD). (**a**) Images of the 2D sample and (**b**) the 3D sample; the atoms are colored according to atomic type and CAN.

The five-element Co–Cr–Cu–Fe–Ni interatomic potential developed by Farkas et al. [39] was used for all the simulations by using the open-source LAMMPS code [40]. Two kinds of atomic models, two-dimensional (2D) sheet and three-dimensional (3D) cubic nano-crystalline samples, were constructed (presented in Figure 1). The size of the 2D HEA sheet in FCC structure with a lattice constant of 3.62 Å [41] was 60 × 60 × 3.62 nm^3^, containing 1,098,730 atoms. The 2D sheet was composed of 16 randomly orientated grains built by the Voronoi methods, except that the crystallographic orientation of the Z axis of each grain was along [0 0 1]. We determined 1,080,458 atoms and 13 randomly orientated grains in the 3D cube with a length of 23.4 nm for every side. In order to achieve equiatomic composition, Fe atoms were randomly selected and replaced with Co (Cr, Cu, Ni). The simulations were conducted using the Nose–Hoover thermostat with the time steps of 1 femtosecond. Periodic boundary conditions were applied in all three directions.

The equilibrium state of the HEA samples could be achieved with the following three steps: first, the as-created Co–Cr–Cu–Fe–Ni HEA samples initially underwent energy minimization; second, the samples were relaxed for 2 ns at the hydrostatic pressure of 5 GPa and 300 K under the NPT (constant number of atoms (N), constant pressure (P), and constant temperature (T)) ensemble; finally, they were relaxed subsequently for 2 ns at the hydrostatic pressure of 0 GPa and 300 K under the NPT ensemble. After reaching equilibrium configurations, uniaxial tensile deformation along the X-direction was applied at 300 K and constant strain rate of 4.0 × 10^7^ s^−1^. In this study, the software OVITO [42] was employed to visualize or analyze the data generated by the MD simulations. The adaptive common-neighbor analysis (CNA) algorithm [43] was used to characterize the local atomic structural environment, in which the green atoms indicate the FCC structure, the blue atoms represent the BCC structure, the red atoms stand for stacking faults, and the white atoms suggest grain boundaries or dislocation cores. Meanwhile, an atomic strain modifier [44] was used to calculate the atomic level deformation gradient; displacement vectors modifier indicated the glide directions of atoms in certain close-packed planes. The local atomic strains were referenced to the relaxed samples before the tensile test.

## 3. Results and Discussion

### 3.1. Random Composition Distribution

**Figure 2:**
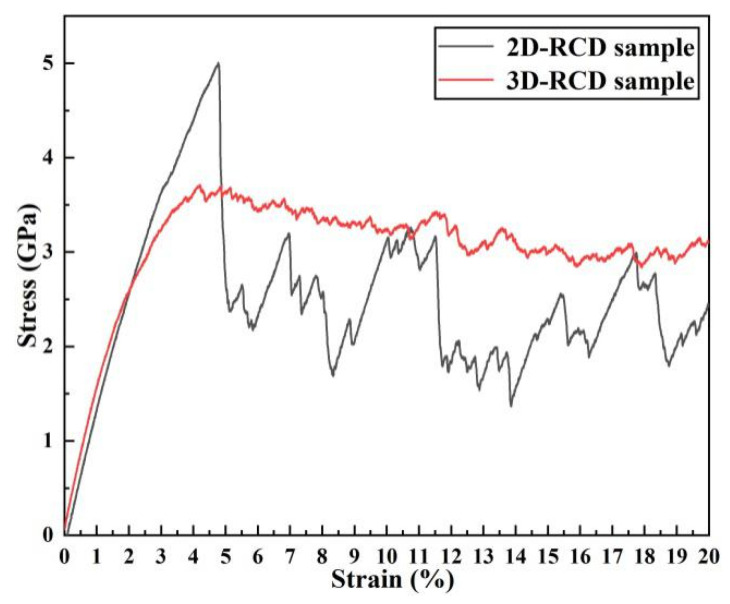
Stress-strain relations for the 2D and 3D RCD samples under uniaxial tensile loading.

**Figure 3:**
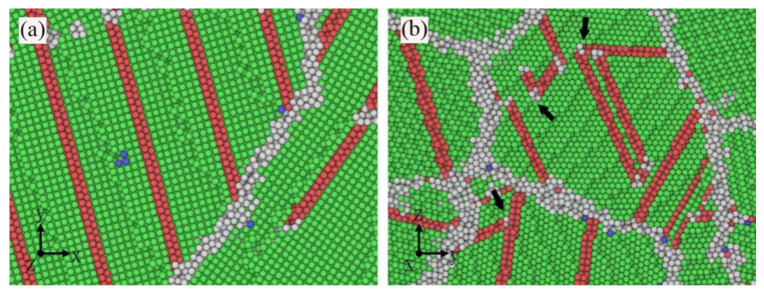
Snapshots of the configurations of the 2D-RCD sample (**a**) and the 3D-RCD sample (**b**) at a strain of 8%.

Figure 2 shows the tensile stress–strain curves of the random composition distribution (RCD) samples at room temperature. In the elastic stage, different grain orientations [45] would cause the elastic modulus of the 2D-RCD sample to deviate from the value of the 3D-RCD sample. The different grain orientations and 3D grain boundary lowered the yield strength of the 3D-RCD sample. At the plastic stage, it was seen that, compared with the 2D-RCD sample, the strain softening resistance of the 3D-RCD sample was better. This was due to the formation of multiple deformation paths in the 3D-RCD sample. The intersections (Figure 3b black arrows) of these plastic slip deformation paths inhibited atoms’ sliding in certain close-packed planes and acted as an obstacle to dislocation motion [46], resulting in a slow drop and small fluctuation of the flow stress of the 3D-RCD sample. As expected, the relatively high loading rate of 4.0 × 10^7^ s^−1^ due to the timescale limitations of MD, the ideal crystal structure, and the extreme purity of the sample led to considerably higher yield stress (in GPa) as compared to that observed in conventional tensile experiments (limited to few hundred MPa) [47,48]. Moreover, the frequent drops and rises of the stress–strain curves were caused by the competition between plastic deformation and strain hardening, which were observed experimentally [47].

**Figure 4:**
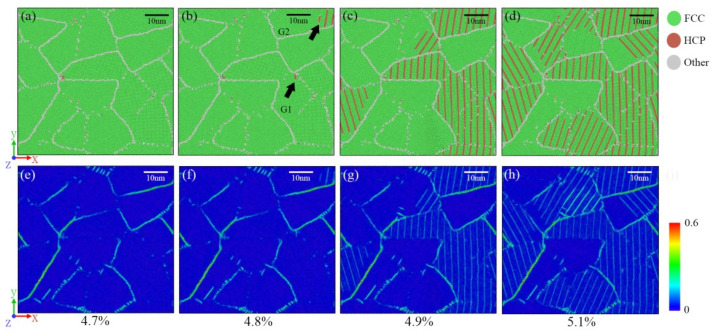
Microstructure evolution (**a**–**d**) and local atomic strain distribution (**e**–**h**) for the 2D-RCD sample at various levels of strain.

To better understand the deformation behavior, the microstructural evolution of the 2D-RCD sample was examined. According to the microstructure evolution visualized by CNA and the atomic strain distribution of the 2D-RCD sample, presented in Figure 4, in the elastic stage (0–4.7%), the strain accumulated mainly in grain boundaries (Figure 4e).

**Figure 5:**
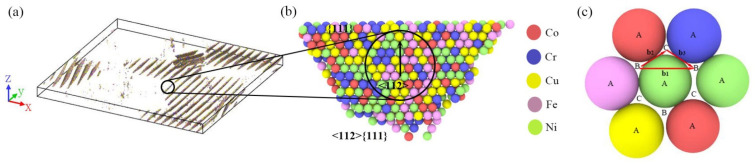
Snapshot of the slip systems and the slip paths of atoms during dislocations’ slip in 2D-RCD samples. (**a**–**c**) Positions of the atoms in different close-packed planes, b_1_ atomic slip path in the case of 1/2<110> full dislocation slip; b_2_ and b_3_ indicate the atomic slip paths in the case of the 1/6<112> partial dislocation slip.

In the plastic stage, due to the different orientation of each grain, the resolved shear stress along the slip direction of the grains, being in a favorable orientation, reached the critical value earlier, and plastic deformation occurred. The sample began to yield, which means that dislocations started to occur and continued along the slip planes, generating double-layer HCP structures in the form of intrinsic stacking fault (ISF) (Figure 4 black arrows). Dislocation slipping is accompanied by grain rotation, which is induced by grain boundary sliding [46]. When the strain increased from 4.8% to 5.1%, the in-plane rotation angles of grains G1 and G2 indicated in Figure 5b were approximately 2.17° and 0.94°, respectively. It indicated that dislocation slipping and grain boundary sliding dominated the primary plastic behavior. When the surrounding grains have different orientations, the dislocations cannot cross the grain boundaries and thus pile up at the grain boundaries [49]. The piling up of the dislocation resulted in high stress, which caused the formation of some slip systems in adjacent grains and other grains with favorable orientation (Figure 4c), thus accommodating the stress and restarting the dislocation of the original grains, as seen in Figure 4d. In this way, plastic deformation was transferred between adjacent grains and finally spread to the entire sample.

To further investigate the characteristics of the slip systems in the grains, we selected the stacking faults from the 2D-RCD sample at a strain of 4.9%; only atoms of the stacking faults are displayed in Figure 5. From the snapshot, it is evident that the activated slip systems in the grains were <112>{111} type (Figure 5b). The reasons why the slip system was <112>{111} rather than the common <110>{111} are as follows: on the one hand, direct slipping along the direction of <110> (Figure 5c-b_1_) resulted in significant collision with the adjacent atom A, causing large local lattice distortion and a significant increase in energy; on the other hand, the lattice distortion caused by the distribution of component atoms with different sizes [48] made slipping along the <110> direction more difficult. Moreover, lattice distortion also reduces stacking fault energy (SFE) [50], which makes it easier for a full dislocation to split into two partials with a wider stacking fault ribbon between them [51]. Therefore, the ideal slip path was along the <112> direction, arriving at position C first (Figure 5c-b_2_) and then getting to the adjacent position B (Figure 5c-b_3_). In a word, the <112>{111} system was more easily activated than the <110>{111} system.

### 3.2. Cu-Rich Grain Boundaries

**Figure 6:**
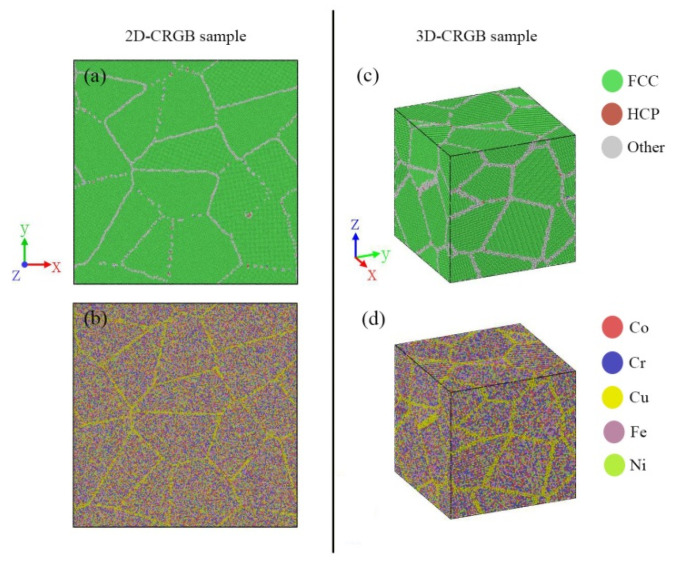
Top views of the 2D-CRGB sample (**a**,**b**) and three-dimensional views of the 3D-CRGB sample (**c**,**d**), the atoms are colored according to CNA and atomic type, respectively.

In order to investigate the effect of composition segregation on the mechanical properties under uniaxial tensile loading and the associated deformation behavior of Co–Cr–Cu–Fe–Ni HEA, a series of MD models were constructed by substituting Cu atoms for Co (Cr, Cu, Fe, Ni) in specified regions (grain boundary or grain) and maintaining the desired equiatomic composition of the whole samples. Therefore, Cu-rich and Cu-depleted regions would separate in the samples. By means of the above method, samples with Cu-rich grain boundaries (CRGB sample, Figure 6) and Cu-rich grains (CRG sample in Section 3.3) were obtained. Moreover, according to the experimental data [16], the Cu content in the Cu-rich regions was as high as 80%.

**Figure 7:**
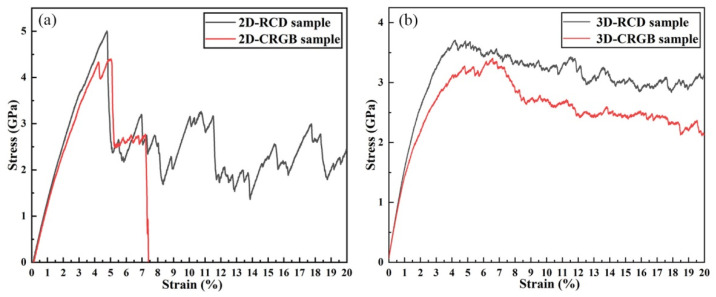
Stress-strain relations of the RCD and CRGB samples under uniaxial tensile loading. (**a**) 2D samples and (**b**) 3D samples.

**Figure 8:**
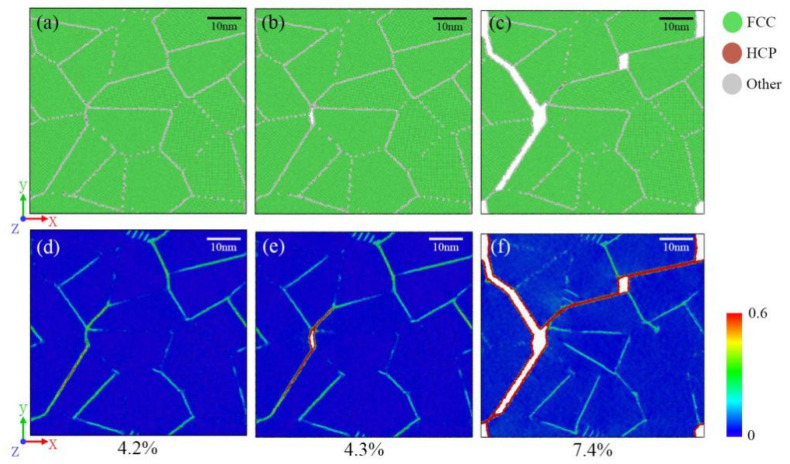
Microstructure evolution (**a**–**c**) and local atomic strain distribution (**d**–**f**) for the 2D-CRGB sample at various strain values.

For Cu-rich grain boundaries, the corresponding stress–strain curves of the 2D and 3D samples are shown in Figure 7. In Figure 7a, it can be observed that, compared with the 2D-RCD sample, the yield strength and plasticity of the 2D-CRGB sample were simultaneously decreased at the constant strain rate of 4.0 × 10^7^ s^−1^. Especially, regarding plasticity, when the strain reached 7.4%, intergranular fracture occurred (see Figure 8c). As for the 3D-CRGB sample, its strength and flow stress obviously decreased compared with those of the 3D-RCD sample (Figure 7b).

Figure 8 shows the microstructure evolution and the local atomic strain distribution of the 2D-CRGB sample. In the elastic stage (0–4.2%), the local atomic shear strain was concentrated on grain boundaries (Figure 8a,d), similar to the elastic deformation of the 2D-RCD sample. However, differently from the 2D-RCD sample, after yielding, a small dislocation slip within grains occurred (Figure 8b,e), and the local stress concentration was relaxed through grain boundary cracking in the 2D-CRGB sample. The reason for this phenomenon may be that, compared with Cu-depleted regions inside the grains, Cu-rich regions at the grain boundaries have a low strength. Furthermore, atoms in grain boundaries are arranged irregularly, which means they have larger potential energy than those in grains [52]. As a result, the grain boundaries cracked before plastic deformation in the grains could relax the local stress concentrations on the grain boundaries.

**Figure 9:**
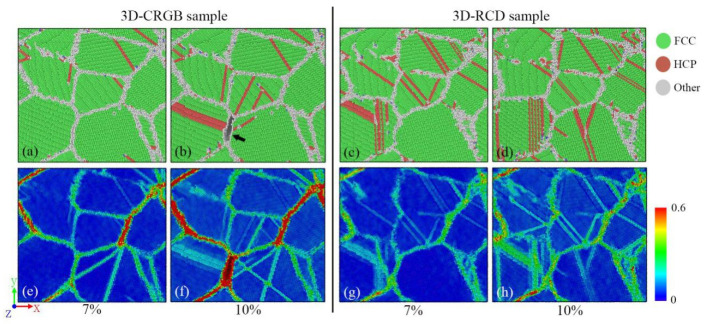
Microstructure evolution (**a**–**d**) and local atomic strain distribution (**e**–**h**) for the 3D-CRGB and 3D-RCD samples at strains of 7% and 10%, respectively.

The microstructure evolution of the 3D-CRGB sample was examined. The results showed that dislocation slip also occurred inside the grains of the 3D-CRGB sample, but the dislocation density was lower than that of the 3D-RCD sample (Figure 9 top panel). Moreover, the local strain was more seriously concentrated on grain boundaries in 3D-CRGB samples (Figure 9 bottom panel), similar to what observed for the 2D samples. This high concentration of strain on the grain boundaries was the reason for the decrease of flow stress in the 3D-CRGB sample. When the strain reached 10%, obvious intergranular fracture occurred in the 3D-CRGB sample (Figure 9 black arrow). The intergranular fracture started at a strain of 7.8%, which led to a sudden decrease in the flow stress of the 3D-CRGB sample.

### 3.3. Cu-Rich Grains

**Figure 10:**
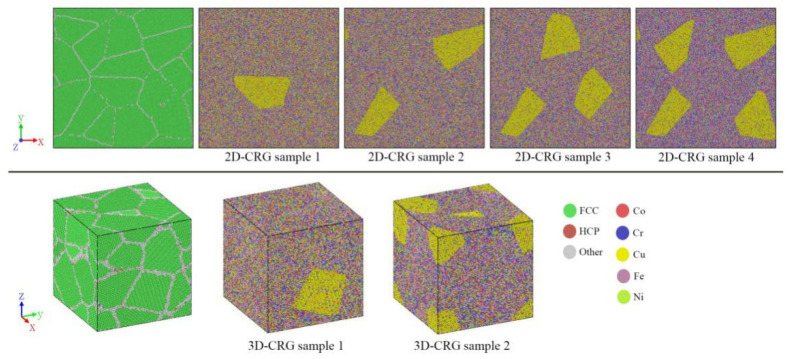
Top views of the 2D-CRG samples and three-dimensional views of the 3D-CRG samples, where the volume fraction of Cu-rich regions in the 2D-CRG samples are 6.7%, 12.3%, 16.7%, 24.4%, and those in the 3D-CRG samples are 9.5% and 15.9%.

**Figure 11:**
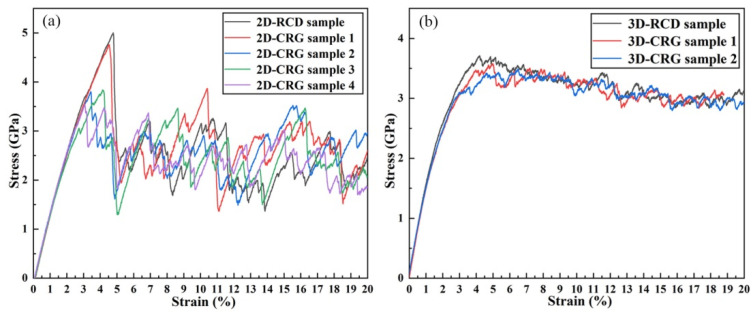
Stress-strain relations for the RCD and CRG samples under uniaxial tensile loading. (**a**) Curves for the 2D samples and (**b**) curves for the 3D samples.

As shown in Figure 10, a series of Cu-rich nanograin samples were constructed. Figure 11a shows the tensile stress–strain curves of the 2D-RCD and CRG samples. As shown in Figure 11a, compared with the 2D-RCD sample, the strength of the 2D-CRG samples was obviously reduced. In addition, the higher the number of Cu-rich grains, the more obvious the decrease of the strength, indicating that the Cu-rich regions distributed in the grains lowered the strength of the samples. This is consistent with previously reported experimental results [18]. As for the 3D-CRG samples, their strength also decreased compared with the 3D-RCD sample (Figure 11b).

**Figure 12:**
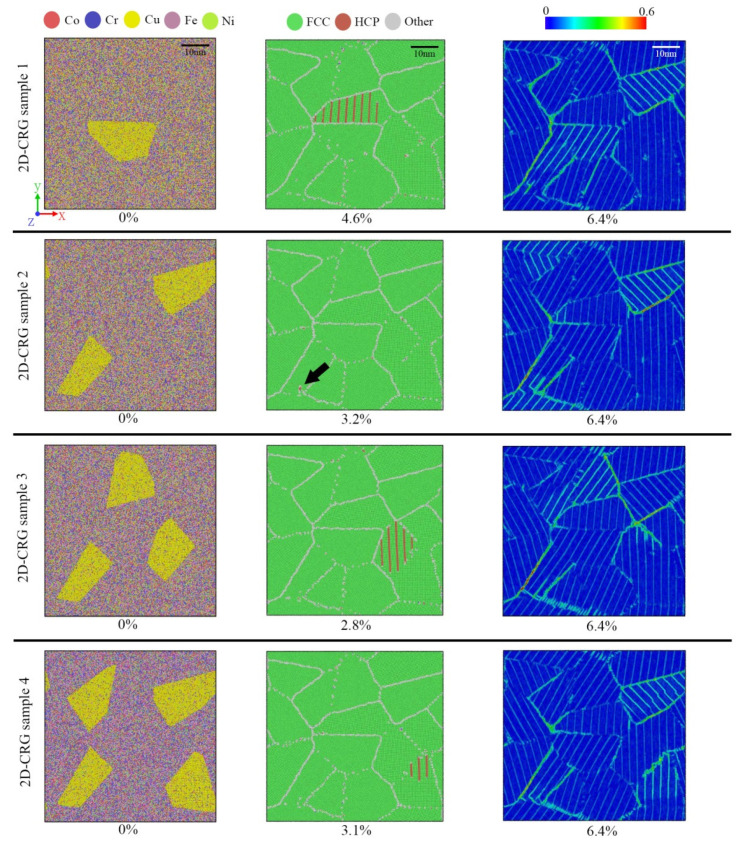
Atomic configurations of 2D-CRG samples at various strains; the atoms are colored according to atomic type, CNA, and local atomic shear strain from left to right.

We also investigated microstructure evolution and local atomic strain distribution for the 2D-CRG samples due to their tensile deformation, presented in Figure 12. Compared with the 2D-RCD sample, except for the 2D-CRG sample 1, the 2D-CRG samples 2–4 yielded from Cu-rich grain, leading to plastic deformation. With the increase of deformation, the strain seemed more concentrated on the Cu-rich regions (Figure 12 right column), different from the local strain distribution of the 2D-RCD sample under the same strain. This was because, compared with the Cu-depleted grains, the strength of the Cu-rich grains was lower. In other words, the yield tended to start from the Cu-rich grain first, which led to a decrease of the strength of the 2D-CRG samples 2–4. As for the CRG sample 1, the yield started from the Cu-depleted grain, which was caused by the uneven stress distribution. Therefore, compared with the 2D-CRG samples 2–4, the strength of the 2D-CRG sample 1 did not decrease significantly (Figure 11b). Detailed explanations are reported below.

In order to further reveal the origin of the deformation behavior of the 2D-CRG samples 1–4, Schmid’s law was adopted. According to Schmid’s law [53], slip systems in grains can only be activated if the resolved shear stresses along the slip directions in the applied stress field are greater than the critical resolved shear stress (CRSS). Therefore, under the condition that the slip system is known, the critical resolved shear stress $\tau_{y}$ can be expressed as $\tau_{y}=\mu \cdot \sigma_{y}$, where μ is the Schmid factor, and $\sigma_{y}$ is the yield strength (GPa).

**Figure 13:**
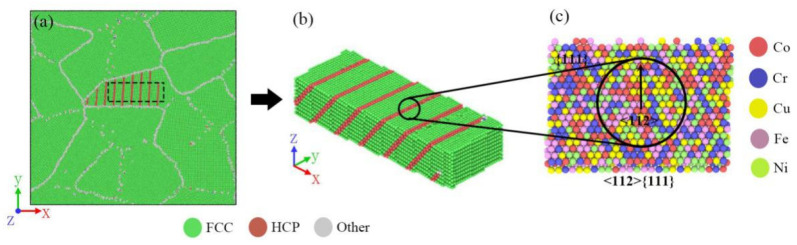
A schematic representation of the calculation of critical resolved shear stress. (**a**) The position of the selected atoms. (**b**)The three-dimensional view of the selected cube. (**c**) The slip systems inside the selected cube.

**Figure 14:**
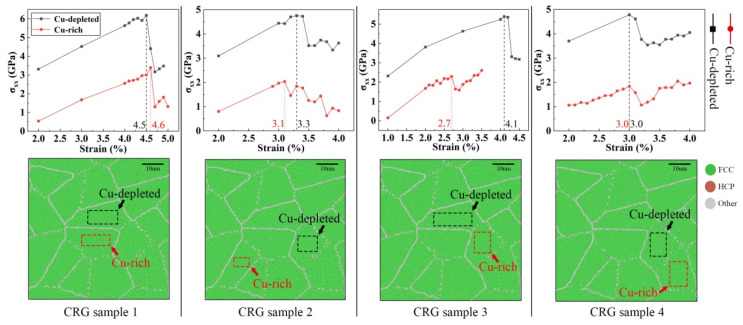
Stress-strain curves (**top panel**) of the cubes (**bottom panel**) obtained from the Cu-rich and Cu-depleted grains of the 2D-CRG samples 1–4.

To provide a clearer description as to the calculation process of CRSS, a schematic illustration is shown in Figure 13. First, atoms in a cube were selected from the inside of a grain (Cu-rich or Cu-depleted), and these selected atoms were tracked during the whole deformation process of the sample (Figure 13a). Cu-rich and Cu-depleted grains where the dislocation slip first occurred in the sample were selected. Second, the stress-strain curves (as shown in the top panel in Figure 14) of the selected cubes (the bottom panel in Figure 14) were calculated to determine the yield stresses of these Cu-rich and Cu-depleted grains. Finally, the slip directions and the slip planes of the slip systems inside the cubes were determined (Figure 13c) to calculate the Schmid factors. Therefore, CRSS could also be calculated. The detailed results are presented in Table 1.

**Figure 15:**
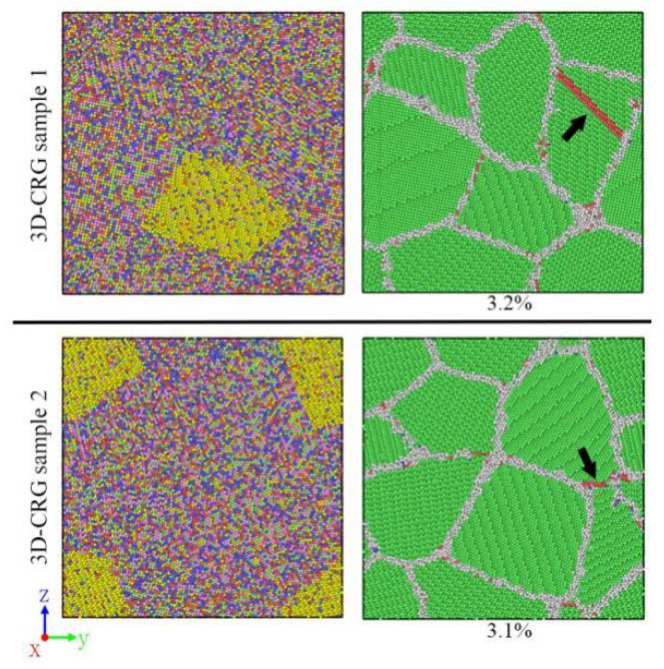
Atomic snapshots showing the dislocations slip from Cu-depleted regions of the 3D-CRG sample at different strains.

It can be clearly seen from Table 1 that the CRSS of the Cu-depleted grains was greater than that of the Cu-rich grains. It means that the slip systems in the Cu-rich grains were more easily activated. In other words, plastic deformations tended to occur in the Cu-rich grains first. Therefore, all the 2D-CRG samples 2–4 yielded from Cu-rich grains (Figure 12). According to Figure 14, the stress–strain curves of Cu-rich grains in the 2D-CRG samples 2–4 reached the yield point first, while in the 2D-CRG sample 1, those of Cu-depleted grains yielded first. This confirmed the yield phenomenon of the 2D-CRG samples 1–4 observed in Figure 12. In addition, it can be seen from the stress–strain curves in Figure 14 that, compared with the stresses received by the Cu-rich grains, the stresses received by the Cu-depleted grains were much higher. This uneven stress distribution made it possible for the sample to yield from the Cu-depleted grain, as observed for the 2D-CRG sample 1 (Figure 12). This is why, compared with the 2D-CRG samples 2–4, the strength of the 2D-CRG sample 1 did not decrease significantly. The results in Table 1 also prove that the strength of the Cu-rich regions was much lower than that of the Cu-depleted regions, which made the macroscopic strength of the 2D-CRGB sample and 2D-CRG samples 1–4 decrease. This is consistent with the results of the tensile tests reported above (Figure 11). Figure 15 shows the atomic snapshots of the 3D-CRG samples 1–2. It was found that the 3D-CRG samples 1–2 yield from Cu-depleted grains, as observed for the 2D-CRG sample 1. Therefore, the decrease in strength of the 3D-CRG samples 1–2 was not as obvious as that of the 2D-CRG samples 2–4 (Figure 11b).

## 4. Conclusions

In summary, we investigated the effect of Cu-rich grain boundaries and Cu-rich grains on the mechanical properties of the Co–Cr–Cu–Fe–Ni HEA. The associated deformation behavior was also studied by using MD simulation. Moreover, a deformation mechanism was presented.

Dislocation slipping and grain boundary sliding were the main deformation mechanisms in the Co–Cr–Cu–Fe–Ni HEA. The internal slip system of the Co–Cr–Cu–Fe–Ni HEA appeared to be mainly of the <112>{111} type. Cu-rich grain boundaries can seriously reduce the plasticity of the Co–Cr–Cu–Fe–Ni HEA, because they can lead to intergranular fracture of CRGB samples. At the same time, the strength of the alloy was slightly reduced by Cu-rich grain boundaries. Cu-rich grains can seriously reduce the strength of the Co–Cr–Cu–Fe–Ni HEA. The larger the volume fraction of Cu-rich grains is, the more obvious the decrease of strength. The reason is that the resolved shear stress required to start slip systems in Cu-rich grains is much smaller than that required in Cu-depleted grains. In other words, plastic deformation tends to occur in Cu-rich grains. Furthermore, an uneven stress distribution was detected, where the stress on Cu-depleted grains was much higher than that on Cu-rich grains. The mechanical properties of the Co–Cr–Cu–Fe–Ni HEA are obviously affected by phase separation, which provides a basis for improving its mechanical properties. This investigation can help to expand the practical application of this kind of alloy.

## Figures and Tables

**Table 1:** Slip system parameters of the 2D-CRG samples 1–4.

Samples	2D-CRG Sample 1		2D-CRG Sample 2		2D-CRG Sample 3		2D-CRG Sample 4	
Region	Cu-depleted	Cu-rich	Cu-depleted	Cu-rich	Cu-depleted	Cu-rich	Cu-depleted	Cu-rich
CRSS	3.01	1.32	2.26	0.7	2.58	1.1	2.26	0.9
μ	0.49	0.39	0.48	0.34	0.48	0.48	0.47	0.49

## Data Availability

The raw/processed data required to reproduce these findings are available from the corresponding author on reasonable request.

## References

[1] Tong C.J., Chen M.R., Chen S.K., Yeh J.W., Shun T.T., Lin S.J., Chang S.Y. (2005). Mechanical performance of the AlxCoCrCuFeNi high-entropy alloy system with multiprincipal elements. Metall. Mater. Trans. A.

[2] Chuang M.H., Tsai M.H., Wang W.R., Lin S.J., Yeh J.W. (2011). Microstructure and wear behavior of Al_x_Co_1.5_CrFeNi_1.5_Ti_y_ high-entropy alloys. Acta Mater..

[3] Yeh J.W. (2013). Alloy Design Strategies and Future Trends in High-Entropy Alloys. JOM.

[4] Zhang Y., Zuo T.T., Tang Z., Gao M.C., Dahmen K.A., Liaw P.K., Lu Z.P. (2014). Microstructures and properties of high-entropy alloys. Prog. Mater. Sci..

[5] Ye Y.F., Wang Q., Lu J., Liu C.T., Yang Y. (2016). High-entropy alloy: Challenges and prospects. Mater. Today.

[6] Li Z.M., Pradeep K.G., Deng Y., Raabe D., Tasan C.C. (2016). Metastable high-entropy dual-phase alloys overcome the strength–ductility trade-off. Nature.

[7] Tsai M.H., Yeh J.W. (2014). High-Entropy Alloys: A Critical Review. Mater. Res. Lett..

[8] Xian X., Lin L.J., Zhong Z.H., Zhang C., Chen C., Song K.J., Cheng J.G., Wu Y. (2018). Precipitation and its strengthening of Cu-rich phase in CrMnFeCoNiCu_x_ high-entropy alloys. Mater. Sci. Eng. A.

[9] Cantor B., Chang I.T.H., Knight P., Vincent A.J.B. (2004). Microstructural development in equiatomic multicomponent alloys. Mater. Sci. Eng. A.

[10] Yeh J.W., Chen S.K., Lin S.J., Gan J.Y., Chin T.S., Shun T.T., Tsau C.H., Chang S.Y. (2004). Nanostructured High-Entropy Alloys with Multiple Principal Elements: Novel Alloy Design Concepts and Outcomes. Adv. Eng. Mater..

[11] Gwalani B., Ayyagari A.V., Choudhuri D., Scharf T., Mukherjee S., Gibson M., Banerjee R. (2018). Microstructure and wear resistance of an intermetallic-based Al_0.25_Ti_0.75_CoCrFeNi high entropy alloy. Mater. Chem. Phys..

[12] Ge W.J., Wu B., Wang S.R., Xu S., Shang C.Y., Zhang Z.T., Wang Y. (2017). Characterization and properties of CuZrAlTiNi high entropy alloy coating obtained by mechanical alloying and vacuum hot pressing sintering. Adv. Powder Technol..

[13] Lu J.B., Wang B.F., Qiu X.K., Peng Z.Q., Ma M.M. (2017). Microstructure evolution and properties of CrCuFe_x_NiTi high-entropy alloy coating by plasma cladding on Q235. Surf. Coat. Technol..

[14] Cheng J.B., Liang X.B., Xu B.S. (2014). Effect of Nb addition on the structure and mechanical behaviors of CoCrCuFeNi high-entropy alloy coatings. Surf. Coat. Technol..

[15] Liu N., Wu P.H., Zhou P.J., Peng Z., Wang X.J., Lu Y.P. (2016). Rapid solidification and liquid-phase separation of undercooled CoCrCuFe_x_Ni high-entropy alloys. Intermetallics.

[16] Verma A., Tarate P., Abhyankar A.C., Mohape M.R., Gowtam D.S., Deshmukh V.P., Shanmugasundaram T. (2019). High temperature wear in CoCrFeNiCu_x_ high entropy alloys: The role of Cu. Scripta Mater..

[17] Wang W.L., Hu L., Luo S.B., Meng L.J., Geng D.L., Wei B. (2016). Liquid phase separation and rapid dendritic growth of high-entropy CoCrCuFeNi alloy. Intermetallics.

[18] Guo T., Li J.S., Wang J., Wang Y., Kou H.C., Niu S.Z. (2017). Liquid-phase separation in undercooled CoCrCuFeNi high entropy alloy. Intermetallics.

[19] Wu P.H., Liu N., Zhou P.J., Peng Z., Du W.D., Wang X.J., Pan Y. (2015). Microstructures and liquid phase separation in multicomponent CoCrCuFeNi high entropy alloys. Mater. Sci. Technol..

[20] Nakagawa Y. (1958). Liquid immiscibility in copper-iron and copper-cobalt systems in the supercooled state. Acta Metall..

[21] Katsumata K., Kameshima Y., Okada K., Yasumori A. (2004). Preparation of phase-separated textures and crystalline phases from two-liquid immiscible melts in the TiO2–SiO2 system. Mater. Res. Bull..

[22] Niu Y.H., Wang Z.G., Orta C.A., Xu D.G., Wang H., Shimizu K., Hsiao B.S., Han C.C. (2007). Acceleration or retardation to crystallization if liquid–liquid phase separation occurs: Studies on a polyolefin blend by SAXS/WAXD, DSC and TEM. Polymer.

[23] Ratke L., Diefenbach S. (1995). Liquid immiscible alloys. Mater. Sci. Eng. R Rep..

[24] Curiotto S., Greco R., Pryds N.H., Johnson E., Battezzati L. (2007). The liquid metastable miscibility gap in Cu-based systems. Fluid Phase Equilibr..

[25] Zhang Y.K., Gao J., Yang C., Kolbe M., Binder S., Herlach D.M. (2012). Asynchronous crystallization behavior of Co-rich droplets in phase-separated Cu–Co alloys. Mater. Lett..

[26] Yamauchi I., Irie T., Sakaguchi H. (2005). Metastable liquid separation in undercooled Fe–Cu and Fe–Cu–Si melts containing a small B concentration and their solidification structure. J. Alloys Compd..

[27] Chen Y.Z., Liu F., Yang G.C., Xu X.Q., Zhou Y.H. (2007). Rapid solidification of bulk undercooled hypoperitectic Fe–Cu alloy. J. Alloys Compd..

[28] Wei X., Wang J.P., Yang Z.M., Sun Z.B., Yu D.M., Song X.P., Ding B.J., Yang S. (2011). Liquid phase separation of Cu–Cr alloys during the vacuum breakdown. J. Alloys Compd..

[29] Munitz A., Bamberger M., Venkert A., Landau P., Abbaschian R. (2009). Phase selection in supercooled Cu–Nb alloys. J. Mater. Sci..

[30] Munitz A., Abbachian R., Cotler C., Shacham C. (1996). Liquid Phase Separation in Cu-Co-Fe and Cu-Fe-Ni-Cr Alloys. High Temp. Mater. Process..

[31] Liu X.J., Zhu J.H., Yang S.Y., Xu W.W., Wang C.P. (2014). Formation of Cu-rich crystalline/NiNb-rich amorphous composite induced by liquid phase separation. Mater. Lett..

[32] He J., Jiang H.X., Chen S., Zhao J.Z., Zhao L. (2011). Liquid phase separation in immiscible Ag–Ni–Nb alloy and formation of crystalline/amorphous composite. J. Non-Cryst. Solids.

[33] Yu Y., Wang C.P., Liu X.J., Kainuma R., Ishida K. (2011). Thermodynamics and kinetics in liquid immiscible Cu–Cr–Si ternary system. Mater. Chem. Phys..

[34] Hsu U.S., Hung U.D., Yeh J.W., Chen S.K., Huang Y.S., Yang C.C. (2007). Alloying behavior of iron, gold and silver in AlCoCrCuNi-based equimolar high-entropy alloys. Mater. Sci. Eng. A.

[35] Derimow N., Abbaschian R. (2018). Solidification microstructures and calculated mixing enthalpies in CoCrCu containing alloys. Mater. Today Commun..

[36] Munitz A., Edry I., Brosh E., Derimow N., MacDonald B.E., Lavernia E.J., Abbaschian R. (2019). Liquid phase separation in AlCrFeNiMo_0.3_ high-entropy alloy. Intermetallics.

[37] Tian Y.Y., Fang Q.H., Li J. (2020). Molecular dynamics simulations for nanoindentation response of nanotwinned FeNiCrCoCu high entropy alloy. Nanotechnology.

[38] Liu J. (2020). Molecular dynamic study of temperature dependence of mechanical properties and plastic inception of CoCrCuFeNi high-entropy alloy. Phys. Lett. A.

[39] Farkas D., Caro A. (2018). Model interatomic potentials and lattice strain in a high-entropy alloy. J. Mater. Res..

[40] Plimpton S. (1995). Fast parallel algorithms for short-range molecular dynamic. J. Comput. Phys..

[41] Praveen S., Murty B.S., Kottada R.S. (2012). Alloying behavior in multi-component AlCoCrCuFe and NiCoCrCuFe high entropy alloys. Mater. Sci. Eng. A.

[42] Stukowski A. (2010). Visualization and analysis of atomistic simulation data with OVITO–the Open Visualization Tool. Model. Simul. Mater. Sci. Eng..

[43] Stukowski A. (2012). Structure identification methods for atomistic simulations of crystalline materials. Model. Simul. Mater. Sci. Eng..

[44] Shimizu F., Ogata S., Li J. (2007). Theory of Shear Banding in Metallic Glasses and Molecular Dynamics Calculations. Mater. Trans..

[45] Chen T.Y., Tan L.Z., Lu Z.Z., Xu H.X. (2017). The effect of grain orientation on nanoindentation behavior of model austenitic alloy Fe-20Cr-25Ni. Acta Mater..

[46] Zhou K., Liu B., Yao Y.J., Zhong K. (2014). Effects of grain size and shape on mechanical properties of nanocrystalline copper investigated by molecular dynamics. Mater. Sci. Eng. A.

[47] Sharma A., Balasubramanian G. (2017). Dislocation dynamics in A_l0.1_CoCrFeNi high-entropy alloy under tensile loading. Intermetallics.

[48] Fang Q.H., Chen Y., Li J., Jiang C., Liu B., Liu Y., Liaw P.K. (2019). Probing the phase transformation and dislocation evolution in dual-phase high-entropy alloys. Int. J. Plast..

[49] Kondo S., Mitsuma T., Shibata N., Ikuhara Y. (2016). Direct observation of individual dislocation interaction processes with grain boundaries. Sci. Adv..

[50] Li J., Chen H.T., Fang Q.H., Jiang C., Liu Y., Liaw P.K. (2020). Unraveling the dislocation–precipitate interactions in high-entropy alloys. Int. J. Plast..

[51] Zhao Y.H., Zhu Y.T., Liao X.Z., Horita Z., Langdon T.G. (2006). Tailoring stacking fault energy for high ductility and high strength in ultrafine grained Cu and its alloy. Appl. Phys. Lett..

[52] Feng S.D., Li L., Chan K.C., Zhao L., Pan S.P., Wang L.M., Liu R.P. (2019). Tuning deformation behavior of Cu_0.5_CoNiCrAl high-entropy alloy via cooling rate gradient: An atomistic study. Intermetallics.

[53] Cai Y., Wu H.A., Luo S.N. (2018). A loading-dependent model of critical resolved shear stress. Int. J. Plast..

